# From Student to Practitioner

**Published:** 1896-09-05

**Authors:** 


### The Hospital

An Institutional Journal of

The Medical Sciences and Hospital Administration, with

AN EXTRA NURSING SUPPLEMENT.

Vol. XX.—No. 519.] [Sept. 5, 1896.

### From Student to Practitioner.

Time was when the finished product of medical education was very superior to the raw material out of which it had been formed. To-day a survey, first of the student and then of the practitioner, raises the question whether the raw material be not superior to the finished product? Certainly the medical professor has reason to be proud of the general intellectual capacity and very considerable attainments in culture of the majority of those who occupy the seats in his lecture hall. It can hardly be said of a considerable proportion of the men in actual practice that they give occasion for pride and satisfaction when they are examined with any sharpness of criticism.

Science is the mother of plain speech. It is not of much use, at any rate in the more scientific callings, such as medicine now is, to be anything but honest and plain-spoken. In speaking of the student of the period let us speak quite plainly. Compared with other young men of his class, with the student of law, or divinity, or with the young man who is destined for commerce, where does the modern medical student stand? He stands, if we are not mistaken, in the front rank. Where will he continue to stand throughout his curriculum? He will for the most part continue to stand in the front rank. For medicine, more than divinity, and probably much more than law, stimulates to hard work, and offers no barriers to the very freest excursions of the mind in all directions. A medical student may push his investigations where he will, the more boldly the better; and neither orthodoxy nor expediency will say him nay. The necessary effect of all this is that before the close of his curriculum the medical undergraduate has become an inquirer, a doubter, an open-eyed traveller on the broadest mental highway in the world. He has also become a man of some mental stamina and strenuousness. His five years of hard work, and his experience in the examination rooms, have developed his powers all round; and he is now a man fit, in many respects, for entering upon almost any independent career

Such is the student during and at the termination of his curriculum of study. What is in store for him in practice? More study, he hopes; deeper investigations; widening knowledge; power to deal with serious cases involving the issues of life and death; an assured social position; moderate financial success; the respect of his fellow-men—these are the visions that loom large and pleasing before him as he nears the gateways of actual practice. It would be a mere platitude to say that many of these hopes may fail to be realised. They most certainly have not been realised in the experience of thousands who are now in practice. What is worth thinking of is the question why they have been realised in the experience of so few? and the further question, how the present generation of students may avoid the rocks on which previous generations have split and bring their vessels to a fairer and more prosperous haven?

It is quite certain that the average student of the present day deserves a better fate than that which has overtaken some previous generations. But if he is to come to a better he must not leave his destiny too much in the hands of other people. There is in medicine what Matthew Arnold in another connection called "a stream of tendency." That stream bears young practitioners in certain well-known directions. They must not allow themselves to "drift" with that stream. On the contrary, it is at the very beginning of practice that they must keep their eyes wide open, their judgment clear, and their resolution firm. These are exactly the points in which large numbers of young medical graduates entirely fail. The newly-fledged doctor is in many respects very much of a child, notwithstanding his M.B. or his M.R.C.S. But this is a fact he will by no means himself admit. Is he not a man of science? Has he not obtained a medal or two, or at least some first-class certificates? All the greater is the pity that for want of a little knowledge of the world and of the practical business thereof he should so often literally "throw himself away."

The young doctor is like the young clergyman, he thinks there is salvation in being in practice on his own account. Too often he finds it is not salvation he has lighted upon in practice, but something which would be better described by the exact opposite of that term. It is here where he fails. For the sake of being in practice for himself he accepts a partnership, or a succession, where the fees are miserable; where, if he is to live at all, he must work so many hours a day that scientific and mental improvement are impossible; where social dignity is out of the question, and where everything he most longed for as a student is entirely and for ever beyond his reach. Or, instead of purchasing, he begins practice *de novo*, taking a small house, putting up his doorplate, and making up his mind to wait. And, as a rule, he does "wait:" wait, until not only is he out at elbows, but his courage is destroyed, his spirit quenched, and his heart broken. How is all this to be avoided? By the exercise of a little commonsense. By the young man's resolving to gain a little experience at other people's expense rather than at his own. A preliminary assistantship of three, four, or five years, not necessarily with the same principal, would, like two or three curacies to the young clergyman, be simply invaluable. But then the assistantship must not be looked upon as a mere method of passing the time, and of being well paid for it. It should be a continuance of studentship, and the methods of study, compulsorily practised at hospital, should now be voluntarily taken up with steady purpose and earnest goodwill. Of course their strenuousness may be a little relaxed. But every day should see its work of study accomplished as well as its work of practice. A few years thus spent teach a young man to know a pitfall when he sees it, and the art of keeping out of pitfalls is one of the most important in all life. The modern medical student is eminently a good fellow, capable, industrious, scientific, honest. We wish for him, above all things, that he may be as happy in his practice as he has been diligent and successful in his studentship. But if he is to be so he must, first and above all things, learn how to take care of himself.

